# Disaster Exposure and Mental Health Among Puerto Rican Youths After Hurricane Maria

**DOI:** 10.1001/jamanetworkopen.2019.2619

**Published:** 2019-04-26

**Authors:** Rosaura Orengo-Aguayo, Regan W. Stewart, Michael A. de Arellano, Joy Lynn Suárez-Kindy, John Young

**Affiliations:** 1 National Crime Victims Research and Treatment Center, Department of Psychiatry and Behavioral Sciences, Medical University of South Carolina, Charleston; 2 Mental Health Disparities and Diversity Program, Department of Psychiatry and Behavioral Sciences, Medical University of South Carolina, Charleston; 3 Department of Psychology, Carlos Albizu University, San Juan, Puerto Rico; 4 Puerto Rico Department of Education, San Juan, Puerto Rico; 5 Department of Psychology, University of Mississippi, Oxford

## Abstract

**Importance:**

Quantifying the magnitude of disaster exposure and trauma-related symptoms among youths is critical for deployment of psychological services in underresourced settings. Hurricane Maria made landfall in Puerto Rico on September 20, 2017, resulting in massive destruction and unprecedented mortality.

**Objective:**

To determine the magnitude of disaster exposure and mental health outcomes among Puerto Rican youths after Hurricane Maria.

**Design, Setting, and Participants:**

Survey study in which a school-based survey was administered to each public school student at all schools in Puerto Rico between February 1 and June 29, 2018 (5-9 months after Hurricane Maria). Of the 226 808 students eligible to participate, 96 108 students completed the survey.

**Main Outcomes and Measures:**

Participants were assessed for exposure to hurricane-related stressors, posttraumatic stress disorder (PTSD), and depressive symptoms, using standardized self-report measures administered in Spanish. Descriptive statistics were compiled for all outcome variables, as was the frequency of individuals reporting clinically elevated symptoms of PTSD or depression. Differences in these statistics across sexes were also examined via *t* tests. Correlations between demographic, geographic, and main outcome variables were also calculated, and regressions were conducted to examine their association with symptoms of PTSD.

**Results:**

A total of 96 108 students participated in the study (42.4% response rate; 50.3% female), representative of grades 3 to 12 across all 7 educational regions of Puerto Rico. As a result of the hurricane, 83.9% of youths saw houses damaged, 57.8% had a friend or family member leave the island, 45.7% reported damage to their own homes, 32.3% experienced shortages of food or water, 29.9% perceived their lives to be at risk, and 16.7% still had no electricity 5 to 9 months after the hurricane. Overall, 7.2% of youths (n = 6900) reported clinically significant symptoms of PTSD; comparison of the frequency of reporting clinically elevated symptoms of PTSD across sex yielded a significant difference (*t* = 12.77; 95% CI of the difference, 0.018-0.025; *P* < .001), with girls (8.2%) exceeding the clinical cutoff score more often than boys (6.1%). Finally, similar analysis of differences in depression between sexes was also significant (*t* = 17.56; 95% CI of the difference, 0.31-0.39; *P* < .001), with girls displaying higher mean (SD) scores (2.72 [3.14]) than boys (2.37 [2.93]). Demographic and risk variables accounted for approximately 20% of variance in symptoms of PTSD (*r*^2^ = 0.195; 95% CI, 0.190-0.200).

**Conclusions and Relevance:**

Survey results indicate that Hurricane Maria exposed Puerto Rican youths to high levels of disaster-related stressors, and youths reported high levels of PTSD and depressive symptoms. Results are currently being used by the Puerto Rico Department of Education to inform targeted and sustainable evidence-based practices aimed at improving mental health outcomes for Puerto Rico’s youths.

## Key Points

**Question:**

What was the magnitude of disaster exposure and mental health outcomes on Puerto Rican youths after Hurricane Maria?

**Findings:**

Results from a public school–based survey administered to 96 108 students revealed that 83.9% saw houses damaged, 57.8% had a friend or family member leave the island, 45.7% reported damage to their own homes, 32.3% experienced shortage of food or water, 29.9% perceived their lives to be at risk, and 16.7% still had no electricity 5 to 9 months after the hurricane. Overall, 7.2% of youths reported clinically significant symptoms of posttraumatic stress disorder, with demographic and risk variables accounting for approximately 20% of variance in symptoms.

**Meaning:**

Puerto Rican youths experienced significant disaster exposure and reported trauma-related symptoms that warrant evidence-based mental health services.

## Teaser

This survey study examines the magnitude of disaster exposure and mental health outcomes among Puerto Rican youths after Hurricane Maria.

## Introduction

On September 20, 2017, Hurricane Maria made landfall in the southeastern part of Puerto Rico, causing catastrophic damage estimated at $90 billion and sustained outages of power, water, and communications.^1^ The measurable effect to the infrastructure and length of time necessary to restore services (ie, 3-13 months) marked the longest basic utility outage in US history. In addition, the hurricane resulted in an estimated 2975 to 4645 deaths.^2,3^ Thus, Hurricane Maria was, and continues to be, a public health crisis for the people of Puerto Rico.

Children are especially vulnerable to the long-term negative outcomes of natural disasters given the disruption to their primary systems of social support (eg, families, communities, and schools).^4^ Although previous studies have indicated that approximately half of children will adjust and recover within 1 year of a natural disaster without intensive intervention, other studies have demonstrated that up to one-third will develop chronic symptoms such as posttraumatic stress, depression, anxiety, substance use, suicidal ideation, and/or aggressive behaviors.^4,5,6,7,8,9,10,11,12,13,14,15,16,17^ In samples of mainland US youths, these symptoms were found to be more pronounced among ethnic minorities.^18,19^

Puerto Rico’s population of children and adolescents just prior to Hurricane Maria was an estimated 657 000, suggesting that a large number of youths could experience mental health difficulties directly attributable to their exposure to this climatic event.^20^ Knowing that the health and educational risks to students were high, the Puerto Rico Department of Education established a program to screen all youths enrolled in public schools for disaster exposure and signs of emotional distress after Hurricane Maria in consultation with trauma researchers familiar with best practices for screening after a natural disaster. Briefly, those best practices are (1) assessment beginning at 3 months after the event, (2) using data to apportion whatever resources exist to the areas of highest need, and (3) measuring symptoms of posttraumatic stress, anxiety, depression, and other known risk factors—for example, demographic characteristics, degree of exposure to disaster, and proximal and sustained stressors as a result of the climatic event.^8,9,11^

This study presents the initial results of this screening effort, which are being used to facilitate the development and wide-scale implementation of an evidence-based system to address the ongoing mental health needs of Puerto Rican youths after Hurricane Maria.^21^ The survey described herein was offered to all public school students in grade 3 or higher in Puerto Rico, representing, to our knowledge, one of the largest postdisaster screening projects in US history and the largest sample of Hispanic youths affected by natural disaster (an understudied group in this literature).^18^ In addition to the best practices cited earlier, the methods were informed by seminal postdisaster studies in Puerto Rico and were culturally and linguistically tailored to the unique social context of the island.^22,23,24,25,26,27^

## Methods

### Participants

A total of 96 108 of 226 808 students participated in the study (42.4% response rate) between February 1 and June 29, 2018 (5-9 months after Hurricane Maria). Of the 92 682 students who reported sex, 46 056 were male (49.7%) and 46 626 were female (50.3%). The sample was reflective of the full range of grades and geographic diversity, with large numbers of participants from every grade and educational region of Puerto Rico (Table 1). Consent was obtained orally, participation was voluntary, and students could opt out of participation at any moment. All surveys were completed anonymously and no identifying information was collected. Written consent was waived by the Puerto Rico Department of Education given that data were deidentified. The procedures were approved by both the Puerto Rico Department of Education and the Medical University of South Carolina Internal Review Board. This study was conducted in accordance with the American Association for Public Opinion Research (AAPOR) reporting guideline.^28^

**Table 1.** Sample Demographics

Demographic	No. (%) of Students (N = 96 108)	% of Regional Population^a^
School grade		
3	1226 (1.3)	NA
4	10 719 (11.2)	NA
5	11 245 (11.7)	NA
6	10 996 (11.4)	NA
7	11 516 (12.0)	NA
8	10 912 (11.4)	NA
9	9243 (9.6)	NA
10	9222 (9.6)	NA
11	9148 (9.5)	NA
12	8066 (8.4)	NA
Unknown or unreported	3815 (4.0)	NA
Educational region		
Arecibo	5666 (5.9)	1.33
Bayamón	13 734 (14.3)	3.34
Caguas	12 194 (12.7)	2.33
Humacao	14 838 (15.4)	3.32
Mayagüez	16 686 (17.4)	3.48
Ponce	17 558 (18.3)	4.34
San Juan	15 432 (16.1)	2.56

Abbreviation: NA, not applicable.

^a^ Percentage of regional population calculated by dividing number of respondents by estimated total population from US Census Bureau data.

### Measures

#### National Child Traumatic Stress Network Hurricane Assessment and Referral Tool

The National Child Traumatic Stress Network Hurricane Assessment and Referral Tool (NCTSN-HART)^6^ was developed by the National Center for Child Traumatic Stress after Hurricane Katrina. The measure’s content is derived in part from the Hurricane-Related Traumatic Experiences–Revised^8,11^ and the University of California Los Angeles Posttraumatic Stress Disorder Reaction Index–Brief (UCLA-RI Brief; R.S. Pynoos and A.M. Steinberg, written communication, May 2018). Collectively, the NCTSN-HART’s items assess exposure to hurricane-related stressors, signs of posttraumatic stress experienced on that basis, and depressive symptoms. Additional items assessing potential protective factors (eg, social support) were added in consultation with postdisaster screening experts. Scoring for exposure to stressor items was constructed to correspond to the original Hurricane-Related Traumatic Experiences–Revised format, which yielded subscales of perceived life threat, actual life threat, loss and disruption, and ongoing loss and disruption (ie, well beyond the immediate physical effect of the hurricane). Similarly, the UCLA-RI Brief was scored according to standards for the brief-format instrument (ie, scores of ≥21 points indicated a likely diagnosis of posttraumatic stress disorder [PTSD] and the need for further evaluation). In addition, the instrument includes 4 items that screen for symptoms of depression. Permission was granted by the authors to implement the NCTSN-HART as a self-report tool after translating it into Spanish and adapting the items for the Puerto Rican context. This adaptation was accomplished through close communication between the Puerto Rico Department of Education and one of us (R.O.-A.), who is native Puerto Rican and bilingual.

#### Distance and Income

Additional calculations were conducted at the level of municipality to discern aerial distance from Hurricane Maria’s landfall in Yabucoa and driving distance from San Juan, which was the central location for relief efforts and disbursement of aid. These values were derived from Google Maps using either Yabucoa or San Juan as the starting point and each individual municipality’s name as the ending point. Distance was incorporated into analyses to examine the association between geography, initial and delayed stressful experiences, and symptoms of posttraumatic stress. Median income for each municipality was obtained from the US Census and also included as a predictor.^20^

### Procedures

Survey implementation was connected to a larger initiative already being implemented by the Puerto Rico Department of Education. Representatives from each public school (N = 1086) received packets and deployment instructions from the Puerto Rico Department of Education detailing survey collection procedures. These school representatives distributed survey packets within their respective school districts, provided instruction to teachers for administration in their classrooms, and were responsible for collecting completed materials and returning them to a central Puerto Rico Department of Education location for processing.

### Statistical Analysis

Descriptive statistics were compiled for all outcome variables, as was the frequency of individuals reporting clinically elevated symptoms of PTSD or depression. Differences in these statistics across sexes were also examined via *t* tests. Correlations between demographic, geographic, and main outcome variables were also calculated, and regressions were conducted to examine their association with symptoms of PTSD. The first of these analyses entailed sex, grade level, and municipality as the first of 2 regression steps, with individual risk factors in the second step. The second analysis involved the same first step, but included municipality median income and distance from hurricane landfall in the second step and individual risk factors in the third and final step. All tests were 2-sided and were conducted using *P* < .05 as the criterion for significance. Given that large samples tend to facilitate statistical significance for even minor discrepancies or associations between groups or variables, the results were also examined in terms of absolute differences. All calculations were conducted using SPSS, version 25.0 (IBM Corporation).

## Results

A total of 226 808 youths were solicited, of whom 96 108 (42.4%) participated. As a result of the hurricane, 83.9% of youths saw houses damaged, 57.8% had a friend or family member leave the island, 45.7% reported damage to their own homes, 32.3% experienced shortages of food or water, and 16.7% still had no electricity 5 to 9 months after the hurricane (Table 2). All results for exposure to hurricane stressors by region are shown in Table 2. Table 3 compiles these items into subscales and presents the other main outcome measures. Inspection of these data indicated that 29.9% of respondents perceived their lives to be at risk during the hurricane, with girls (34.2%) reporting this experience significantly more often than boys (27.3%; *t* = 22.65; 95% CI of the difference, 0.06-0.07; *P* < .001). Mean scores on the UCLA-RI Brief were not in the clinical range of elevation (as expected), but did differ by sex (*t* = 27.69; 95% CI of the difference, 1.18-1.35; *P* < .001), with girls exhibiting higher mean (SD) scores (9.29 [7.16]) than boys (8.03 [6.76]). Overall, 7.2% of children (n = 6900) reported clinically significant symptoms of PTSD; comparison of the frequency of reporting clinically elevated symptoms of PTSD across sex yielded a significant difference (*t* = 12.77; 95% CI of the difference, 0.018-0.025; *P* < .001), with girls (8.2%) exceeding the clinical cutoff score more often than boys (6.1%). Finally, similar analysis of differences in depression between sexes was also significant (*t* = 17.56; 95% CI of the difference, 0.31-0.39; *P* < .001), with girls displaying higher mean (SD) scores (2.72 [3.14]) than boys (2.37 [2.93]).

**Table 2.** Percentage of Sample by Educational Region Reporting Exposure to Hurricane Stressors

Item Description	Youths, No. (%)							
	Overall (N = 96 108)	Arecibo (n = 5666)	Bayamón (n = 13 734)	Caguas (n = 12 194)	Humacao (n = 14 838)	Mayagüez (n = 16 686)	Ponce (n = 17 558)	San Juan (n = 15 432)
Injured during hurricane	3895 (4.1)	189 (3.3)	643 (4.7)	479 (3.9)	723 (4.9)	575 (3.4)	622 (3.5)	664 (4.3)
Family, friend, or neighbor injured during hurricane	15 367 (16.0)	835 (14.7)	2397 (17.5)	1983 (16.3)	2444 (16.5)	2511 (15.0)	2612 (14.9)	2585 (16.8)
Family, friend, or neighbor died	6376 (6.6)	366 (6.5)	933 (6.8)	823 (6.7)	1076 (7.3)	1134 (6.8)	1135 (6.5)	909 (5.9)
Thought own life was at risk	28 729 (29.9)	1505 (26.6)	4090 (29.8)	3918 (32.1)	5147 (34.7)	4321 (25.9)	5068 (28.9)	4680 (30.3)
Own house damaged	43 901 (45.7)	2333 (41.2)	6958 (50.7)	6303 (51.7)	7841 (52.8)	6108 (36.6)	7264 (41.4)	7094 (46.0)
Belongings damaged	29 818 (31.0)	1433 (25.3)	4620 (33.6)	4200 (34.4)	5538 (37.3)	3893 (23.3)	5001 (28.5)	5133 (33.3)
Forced to evacuate	24 537 (25.5)	1381 (24.4)	3743 (27.3)	2894 (23.7)	3396 (22.9)	4510 (27.0)	5166 (29.4)	3447 (22.3)
Still relocated	5284 (5.5)	340 (6.0)	941 (6.9)	755 (6.2)	931 (6.3)	699 (4.2)	914 (5.2)	704 (4.6)
Saw houses damaged	80 608 (83.9)	4748 (83.8)	11 717 (85.3)	10 458 (85.8)	12 513 (84.3)	13 507 (80.9)	15 000 (85.4)	12 665 (82.1)
Lost a pet	7212 (7.5)	467 (8.2)	1061 (7.7)	1000 (8.2)	1286 (8.7)	1144 (6.9)	1218 (6.9)	1036 (6.7)
Shortage of food or water	31 086 (32.3)	1997 (35.2)	4747 (34.6)	3979 (32.6)	4663 (31.4)	5138 (30.8)	5601 (31.9)	4961 (32.1)
Theft in neighborhood	16 998 (17.7)	1082 (19.1)	2285 (16.6)	2307 (18.9)	3081 (20.8)	2730 (16.4)	2975 (16.9)	2538 (16.4)
Violence in neighborhood	11 990 (12.5)	571 (10.1)	1780 (13.0)	1601 (13.1)	2073 (14.0)	1638 (9.8)	1842 (10.5)	2485 (16.1)
Moved schools	5474 (5.7)	249 (4.4)	604 (4.4)	805 (6.6)	746 (5.0)	1161 (7.0)	713 (4.1)	1196 (7.8)
Helped rescue people	23 124 (24.1)	1250 (22.1)	3622 (26.4)	2986 (24.5)	3371 (22.7)	3795 (22.7)	4366 (24.9)	3734 (24.2)
Parent(s) lost job	10 964 (11.4)	677 (11.9)	1583 (11.5)	1511 (12.4)	2072 (14.0)	1586 (9.5)	1494 (8.5)	2041 (13.2)
Electricity unrestored	80 020 (16.7)	747 (13.2)	3307 (24.1)	2381 (19.5)	5091 (34.3)	1025 (6.1)	2348 (13.4)	1189 (7.7)
Water unrestored	6995 (7.3)	455 (8.0)	1173 (8.5)	750 (6.2)	1161 (7.8)	1223 (7.3)	1140 (6.5)	1093 (7.1)
Friends or family left island	55 550 (57.8)	3219 (56.8)	8053 (58.6)	7381 (60.5)	9351 (63.0)	9052 (54.2)	9448 (53.8)	9046 (58.6)

**Table 3.** Summary of Outcome Variables

Scale Description	Score, Mean (SD)		
	Overall (N = 96 108)	Boys (n = 46 056)	Girls (n = 46 626)
Perceived risk to life (range, 0-1)	0.30 (0.46)	0.27 (0.44)	0.33 (0.47)
Tangible risk to life (range, 0-6)	1.03 (1.02)	1.02 (1.03)	1.06 (1.01)
Loss and disruption (range, 0-8)	2.20 (1.36)	2.15 (1.37)	2.25 (1.34)
Ongoing loss (range, 0-4)	0.87 (0.74)	0.85 (0.75)	0.89 (0.73)
Total risk (range, 0-19)	4.41 (2.44)	4.29 (2.48)	4.53 (2.40)
Depression (range, 0-16)	2.55 (3.05)	2.37 (2.93)	2.72 (3.15)
UCLA-RI Brief	8.65 (7.00)	8.03 (6.76)	9.29 (7.16)
Elevated symptoms of PTSD, No. (%)	6901 (7.2)	2815 (6.1)	3860 (8.3)

Abbreviations: PTSD, posttraumatic stress disorder; UCLA-RI, University of California Los Angeles Posttraumatic Stress Disorder Reaction Index.

Correlations among the variables of interest appear in Table 4, including correlations between exposure to stressors, symptoms of PTSD, and geographic variables calculated to estimate the distance between landfall, relief supplies, and individual municipalities. Exposure to hurricane-related stressors was moderately correlated with PTSD symptoms. Ongoing loss and disruption, however, was not. In addition, no geographical variable exhibited a meaningful association with other variables of interest (significant correlations were on the basis of sample size). The association between median income and clinical variables was similarly inconsequential; however, a moderate negative correlation between income and distance variables suggested that higher-income areas were (on average) among the closest to both landfall and later aid coordinated through San Juan, Puerto Rico’s capitol.

**Table 4.** Correlations Among Variables^a^

Variable	Median Income	Landfall	Aid	Perceived Risk to Life	Actual Risk to Life	Loss or Disruption	Ongoing Loss or Disruption	Depression	PTSD
Median income	NA	−0.48	−0.66	0.01	0.03	0.03	−0.03	0.02	0.02
Landfall	NA	NA	0.77	−0.06	−0.07	−0.06	−0.14	−0.06	−0.04
Aid	NA	NA	NA	−0.04	−0.07	−0.06	−0.09	−0.04	−0.03
Perceived risk to life	NA	NA	NA	NA	0.20	0.25	0.08	0.20	0.32
Actual risk to life	NA	NA	NA	NA	NA	0.41	0.17	0.22	0.28
Loss or disruption	NA	NA	NA	NA	NA	NA	0.19	0.27	0.34
Ongoing loss or disruption	NA	NA	NA	NA	NA	NA	NA	0.12	0.11
Depression	NA	NA	NA	NA	NA	NA	NA	NA	0.64
PTSD	NA	NA	NA	NA	NA	NA	NA	NA	NA

Abbreviations: NA, not applicable; PTSD, posttraumatic stress disorder.

^a^ All values of the correlation coefficient *r*^2^ are statistically significant (*P* < .001).

Finally, a regression analysis was conducted to examine demographic and risk variables in terms of their association with PTSD symptoms. Sex, school grade, and municipality were entered as the first step in this analysis, and individual risk subscales were entered simultaneously as the second step. The results of this regression were significant (*F* = 281.48; *P* < .001) and accounted for approximately 20% of variance in symptoms of PTSD (*r*^2^ = 0.195; 95% CI, 0.190-0.200), almost all of which was attributable to risk variables entered in the second step. Repeating this analysis with median income and geographical distance from landfall and aid entered as the second step, with risk variables moved to a third step, did not materially change the results (overall *r*^2^ = 0.196).

## Discussion

The main findings of the study indicated that youths in Puerto Rico experienced significant disaster-related exposures as a result of Hurricane Maria. Unlike most disasters or negative etiologic risk factors, however, this devastation and concomitant child and adolescent mental health impairment appeared to be nearly ubiquitous regardless of geographical location or socioeconomic status. In addition to the direct effects of the hurricane, the subsequent implications for Puerto Rico’s economy, culture, and rebuilding efforts were compounded given the mass exodus of much of its populace.^29,30^ This expatriation is reflected in the current data in that 57.8% of respondents reported having a friend or family member who moved away from the island after the hurricane, representing substantial social upheaval and the need for individual adjustment.

Overall results also indicated broad exposure to numerous stressful characteristics associated with the hurricane, including witnessing one’s home and other homes being damaged; having belongings damaged; being forced to evacuate; having a family, friend, or neighbor experience injury or die; or fearing death or injury of self. In addition, children also reported numerous stressors associated with the aftermath of the storm, including shortages of food and water, theft and violence in neighborhoods, and friends or family leaving the island. Based on the results of the screening for traumatic stress symptoms, 7.2% of children would likely have a diagnosis of PTSD at the time data were collected for this study. This latter finding was disproportionately present for girls, which is consistent with previous examinations comparing rates of posttraumatic symptoms across sexes.^31,32^ Initial loss, social disruption, and fear for one’s life (whether perceived or objective) were associated with contemporaneous symptoms of PTSD, but long-term disruptions to resources were not. This finding suggests adaptation to disrupted and impoverished circumstances, unprecedented in US history prior to this point, and resilience on the part of the Puerto Rican people in facing this adversity.

It is also possible to further contextualize the results of this study in comparison with previous examinations of children and adolescents subsequent to a natural disaster in Puerto Rico. For example, a study of the association of Hurricane Georges (which made landfall in Puerto Rico on September 21, 1998) with child and adolescent mental health yielded similar rates of exposure to potentially traumatic events and hurricane-related stressors, but much lower rates of likely diagnoses of PTSD (0.8% vs 7.2% in the current study).^24^ These discrepancies could be attributable to differences in the magnitude of the storms, given that Hurricane Georges was a category 3 storm and Hurricane Maria was a category 4 storm. In addition, the previous study was conducted approximately 18 months after the hurricane, whereas the current surveys were administered between 5 and 9 months after Hurricane Maria. It is possible that the elapsed time between disaster and measurement of the former study contributed to remission of symptoms in many youths who would have been categorized as having a likely diagnosis of PTSD. Evidence suggests that this pattern of gradual remission has a pronounced escalation between 8 and 15 months after a disaster, wherein measurably 50% of diagnoses of PTSD dissipate.^12^ These differences are unlikely to account for the entire range of differences in diagnoses of PTSD, however, given that even liberal application of these data on diagnostic trajectories would estimate an approximately 2% base rate of PTSD by 8 months after Hurricane Georges in the former study.^24^

Comparison with studies conducted after other mainland US disasters, however, indicated that the rates of likely PTSD observed in the current study were lower than those typically reported (13%-30%).^8,12,33^ Similar to the trends in trajectory of diagnoses of PTSD noted earlier, the consensus findings of these studies indicated a steep rate of remission beyond 8 months after the disaster. Although direct comparisons across studies conducted at different times and in different contexts are not fully informative, the differences noted in the base rates of PTSD are suggestive of some interpretations. In particular, the fact that likely diagnoses of PTSD were much less common in the current data (despite findings of extreme devastation, prolonged impairment to the infrastructure, and one of the highest mortality rates among all natural disasters in US history) confers the possibility of moderating factors between traumatic exposure and the eventual development of symptoms of PTSD that were not assessed in the current study. For example, biological differences have been previously noted to be associated with differential responses to traumatic events, and the possibility of sociocultural factors such as *familismo*, a cultural value placing importance on strong family ties, may play a role in a given population’s collective response to adversity and confer potential resilience.^34^ The lack of inclusion of such potential moderating factors is a limitation of this study.

### Limitations

Another potential study limitation is the lack of validation of the Spanish translation of the NCTSN-HART measure, which was not validated in Spanish at the time of the study and had not been previously implemented in Puerto Rico. Although the performance of this instrument in its other applications has been noted to be psychometrically strong, future studies should nonetheless examine whether these findings hold for studies in Puerto Rican or other Spanish-speaking samples. Additional limitations include a somewhat narrow focus on PTSD and depression, given that traumatic exposure could have had a broader association with many more areas of youths’ lives. This was unfortunately a calculated trade-off in the design of this study, particularly considering the costs and attempts to move quickly in data collection to help prioritize deployment of resources to schools (eg, personnel trained in providing evidence-based, trauma-informed practices). In addition, the lack of predisaster data on these same children and adolescents (inherent in nearly all disaster-related research) limited the ability to discern a more precise and/or individualized effect of the hurricane. Given the thorough response of the Puerto Rico Department of Education and greater public recognition of the association of disasters with mental health, it is possible that policy development may eventually allow future studies to address this limitation by facilitating annual, identifiable completion of mental health surveys by all students every school year.

## Conclusions

Despite the noted limitations, this study contributes to the knowledge of the magnitude of natural disasters and mental health symptoms of Hispanic youths and broadens the scope of investigations by including a large population of public school–based children and adolescents. It is also illustrative of the benefits of research and applied partnerships in responding to social and public system difficulties.^35,36^ The work described herein began when the Puerto Rico Department of Education approached Medical University of South Carolina personnel the day after Hurricane Maria had dissipated, and was constructed from the onset to entail a community-based participatory approach.^36,37,38^ Ongoing collaboration is also explicitly intended, which will, we hope, enable infusion of science into practice through development of an evidence-based system and eventual diffusion into the fabric of typical educational procedures.^21^ In particular, the Puerto Rico Department of Education is currently using the findings of this work to most efficiently and effectively deploy aforementioned trauma-focused resources to the areas of greatest need. This deployment is being accomplished through continued collaboration in the form of Medical University of South Carolina personnel along with island- and mainland-based partners providing training, ongoing monitoring, consultation in trauma-informed care, and resilience-building training to school teachers, social workers, and psychologists, who in turn will facilitate future research collaborations in several areas through their provision of services to children and adolescents in need (eg, methods of training, implementation factors, and treatment outcome). This reciprocal cooperation and administration of both research and clinical services is also being conducted with very little budget, thus necessitating creativity, communication, and careful planning of all parties involved. The results of this needs assessment are helping inform targeted and sustainable evidence-based practices aimed at improving mental health outcomes for Puerto Rico’s youths after Hurricane Maria.
